# Effects of laser acupuncture on anthropometric parameters and lipid profile in obese adolescents

**DOI:** 10.1007/s10103-023-03861-8

**Published:** 2023-09-05

**Authors:** Rasha Zohdy, Jehan Alsharnoubi, Wafaa Kandeel, Maha Saber, Hanaa Reyad Abdallah Elmorsy, Ola Dabbous

**Affiliations:** 1 Biological Anthropology, Medical Research and Clinical Studies Institute, National Research Centre, Cairo, Egypt; 2 Pediatrics, National Institute of Laser Enhanced Sciences (N.I.L.E.S), Cairo University, Giza, House 2 street 6 Zahraa Helwan, Cairo, Egypt; 3 Biological Anthropology, Medical Research and Clinical Studies Institute, National Research center, Cairo, Egypt; 4 Childhood Health Complementary Medicine Department, Medical Research and Clinical Studies Institute, National Research Center, Cairo, Egypt; 5 Medical Application of Laser, National Institute of Laser Enhanced Sciences (N.I.L.E.S), Cairo University, Cairo, Egypt

**Keywords:** Laser acupuncture, Anthropometric parameters, Lipid profile, Obesity, Adolescents

## Abstract

The purpose was to compare the effects of diet and exercise and laser interference and maneuver on anthropometric parameters and blood effects of object agents. The study was a randomized controlled longitudinal study. It included 45 adolescents from both sexes who were divided randomly into two groups: one group was treated with low calorie diet and exercise as group A and group B was treated with low calorie diet, exercise, and laser acupuncture. All parameters and blood samples were done before and after the interventions. Group A showed a highly significant reduction post intervention in anthropometric parameters: triceps skin fold thickness (SFT), biceps SFT, subscapular SFT, suprailiac SFT, abdominal SFT, mid-upper arm circumference (MUAC), waist C, and hip C, except for waste/hip (W/H) ratio which showed no significant difference**,** *p* > 0.05 and some lipid profiles (cholesterol, LDL) with *p* < 0.001 Whereas, a significant decrease in TG was observed (*p* < 0.05). On the other hand, a significant increase in HDL was observed (*p* < 0.05). Group B (LCD + exercise + LA) showed a highly significant reduction post intervention in anthropometric parameters: triceps SFT, biceps SFT, subscapular SFT, suprailiac SFT, abdominal SFT, MUAC, waist C, and Hip C, except for W/H ratio which showed no significant difference**,** *p* > 0.05 and lipid profile: cholesterol, LDL, and TG with (*p* < 0.001), whereas a highly significant increase in HDL was observed (*p* < 0.001). The current study revealed a significant difference between group A and group B regarding the percentage of change, where the highest values were found in group B compared to group A, in anthropometric parameters (weight, BMI, subscapular SFT, MUAC, waist circumference) and some lipid profiles (LDL and HDL) with *p* < 0.05. Laser acupuncture was a safe, easy, and more effective tool with extra effect in management of obesity when added to diet and exercise on anthropometric parameters and lipid profile.

## Introduction

Adolescence, a very important stage of human growth and development, is characterized by dynamic physical growth and maturation of secondary sexual characteristics [1]. It was declared by WHO (2022) in World Obesity Day 2022 that 340 million adolescents and 39 million children are obese worldwide [2]. Body mass index (BMI) between the 85th and 94th percentiles is considered to be overweight according to the Centers for Disease Control and Prevention (CDC), while obesity is defined as having a BMI that is above the 95th percentile for both gender and age [3]*.* Due to the increase of dietary energy availability in Egypt, a nutrition shift has been occurring. Overweight and obesity are strongly linked with specific types of diets, including high consumption of fats, animal-based meals, and processed foods [4]***.*** Studies have revealed that obesity is associated with higher circulating concentrations of inflammatory cytokines than in lean individuals. In this way, blood concentrations of these cytokines are lowered following weight loss. The main cytokines responsible of chronic inflammation are tumor necrosis factor-α (TNFα), interleukin-6 (IL-6), and IL-1β. Moreover***,*** such inflammatory cytokines are believed to play a role in insulin resistance [5]***.*** Although several approaches can be applied to prevent and treat adolescent obesity; for example, dietary therapy, therapeutic exercise, and behavior modifications, yet these approaches are difficult for adolescents to undertake [6]***.*** Acupuncture has demonstrated effective therapeutic outcomes in the management of obesity, and laser acupuncture (LA) has the potential to help obese people lose weight and lower their BMI [7]. In laser acupuncture, laser stimulates acupuncture points; the main advantages of laser acupuncture are easy application, precision of dose measurement, being painlessness, and non-invasiveness, being quick, safe, inexpensive, and has no risk of infection [8]***.*** The laser acupuncture’s photobiomodulation impact enhances local vasodilatation and boosts blood flow. This improvement lessens neuro-inflammation, tissue damage, and pro inflammatory cytokines like interleukin-6 (IL-6) and tumor necrosis factor-alpha (TNF-alpha) [9].

## Materials and methods

### Patients

A total of 45 obese adolescents from both sexes with body mass index ≥ the 95^th^ percentile [10] were selected from the Complementary Medicine Clinic at Medical Research Centre of Excellence in the National Research Centre (NRC) and pediatrics clinic at National Institute of Laser Enhanced Sciences.

The protocol was approved by the “Ethical Committee” of the “National Research Centre.” The agreement reference number is 15/104. Then, informed consents were obtained from the parents after the explanation of the aim of the study.

### Design of study

This is a randomized controlled study, in which the subjects were divided randomly and allocated with a computer. So, the total 45 subjects were divided into two groups according to type of treatment by using a computerized program (MedCalc©) in ratio 1:2 as follows: group A comprised 15 obese adolescents who were treated with low calorie diet and exercise with odd numbers and group B comprised 30 obese adolescents who were treated with low calorie diet, exercise, and laser acupuncture with even numbers for 12 weeks.

Inclusion criteria: adolescents with ages 14–18 years. Body mass index ≥ the 95^th^ percentile for adolescents according to the Egyptian growth curves for adolescents.

Exclusion criteria: medical history of chronic diseases, e.g., cardiovascular, respiratory, renal, hepatic or endocrinal as thyroid and adrenal disorders, depression and eating disorders as binge eating, and genetic syndromes as Prader-Will. Adolescents used medications associated with weight gain, e.g., oral antidepressants and long-term oral steroids and use of a pacemaker.

### Anthropometric evaluations

**Fig. 1:**
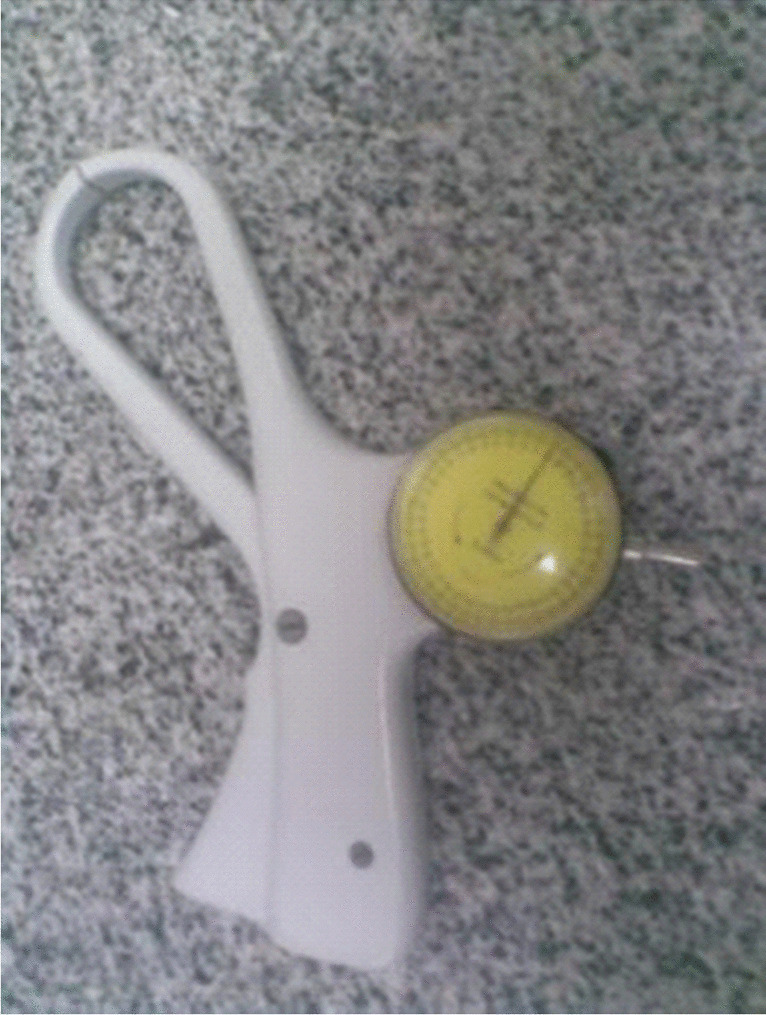
Holtain Tanner/Whitehouse Skinfold Caliper

The subjects were conducted both before and after management. Following the guidelines of the International Biological Program, the height, weight, and skin fold thicknesses at 5 sites (triceps, biceps, subscapular, suprailiac, and abdomen) were assessed [11]. Confirming the subject dressed minimal clothes and without shoes, the subject’s height was measured with a Stadiometer portable scale to the nearest 0.1 cm, and their combined weight was calculated to the nearest 0.01 kg. For the purpose of choosing the sample, the body mass index (BMI), which is calculated as weight (in kilograms) divided by height (in meters squared), was determined. The Holtain skin fold caliper (Fig. 1) was used to measure the skin fold thicknesses, which were then estimated to within 0.1 mm. Additionally, using non-elastic measuring tape, the circumference of the waist, hips, and mid-upper arms was measured to the nearest 0.1 cm.

### Diet and exercise interventions

Both groups followed a healthy, low-calorie diet as well as five to six sessions per week of regular, moderate cardiovascular activity (brisk walking), 30 min/time, 150 min/week. Diets were tailored to each person’s unique calorie needs in order to attain a 500 kcal/person/day deficit. It recommended high-fiber, low-glycaemic index meals with 55% of calories coming from carbohydrates, 25% from fat, and 20% from protein [12]. The main daily food items prescribed were boiled egg, low-fat milk and dairy products, broad bean dip (Ful medames), steamed and fresh vegetables, fruits, whole grains product, low-fat meat, chicken, and fish which were either boiled or roasted. Green tea, coffee, cinnamon, and natural fresh fruit were recommended as beverages. All sugar sweets and carbonate beverages were prohibited. It was done under the supervision of a clinical nutrition consultant and was followed to assess the impact of a dietary behavior modification intervention to reach the ideal weight for age and sex. Nutritional education and behavior modification were performed first. They underwent an identical dietary monitoring program, with an initial consultation, a check-up in the middle of the program, and another during the final sessions by a dietician who was blinded to the type of the program that the subject had been following.

### Laser acupuncture

**Fig. 2:**
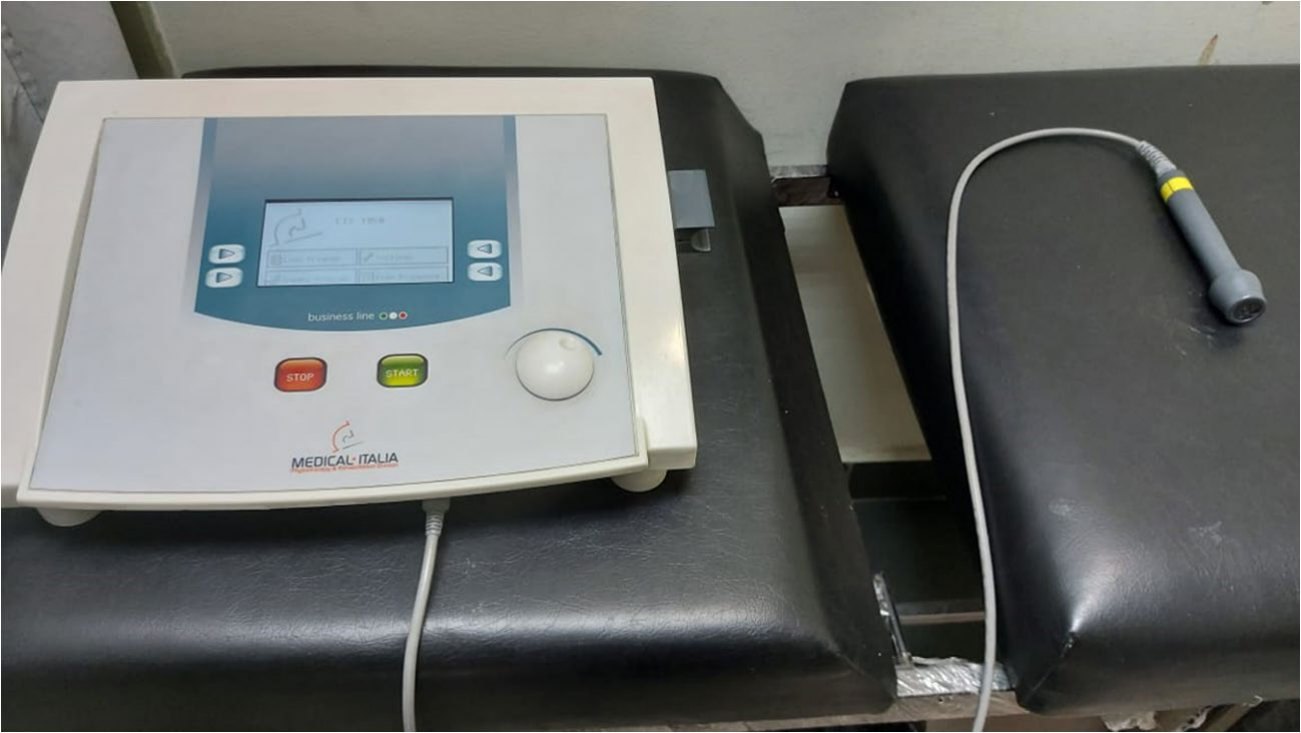
Diode laser device (Medical Italia n. LIS 1050) for laser acupuncture

Subjects received activated laser acupuncture applied by a GaAlAs semiconductor diode laser phototherapy device (Medical Italia n. LIS 1050) (Fig. 2), with parameters listed in Table 1. Laser was applied 2 times a week for 12 weeks. It was applied on the following acupoints: ST25 (Tianshu), ST36 (Zusanli), ST40 (Fenglong), ST44 (Neiting), LI4 (Hegu), LI11 (Quchi), SP6 (Sanyinjiao), and PC6 (Neiguan) bilaterally 1 min for each point. Stimulation of the QuChi (LI 11) and Tianshu (St 25) body acupuncture points has a regulatory effect on intestinal motility**,** whereas the stimulation of Zusanli (St 36) and Neiting (St 44) increases excitability of the satiety center in the ventral medial nucleus of the hypothalamus [8].

**Table 1:** Laser parameters used in group B

1. Wavelength 905 nm	
2. Number of treatments: 2 times a week for 12 weeks	
3. Irradiation time: 30 s (1 min for both point bilateral)	
4. Power output: 100 mw	
5. Energy: 6 J/cm^2^, frequency: 10,000 Hz	
6. Continuous mode	
7. Probe: MLA1/100	
8. It was applied on the following acupoints: ST25, ST36, ST40, ST44, LI4, LI11, SP6, and PC6 [8]***,*** bilaterally	

### Biochemical assessment

We took 5 mL of venous blood from participants after 12 h fasting. After clotting, the blood samples were centrifuged, and the serum will be separated and kept at −80 °C for the assay of lipid profile including total cholesterol, HDL, LDL, and triglycerides assessed by the spectrophotometric method. These samples were taken before and after interventions.

### Sample size

Based on Hulley et al. [13], the standard deviations of the differences were calculated to be 5.56 and 7.33 for groups A and B, respectively. So the effect sizes were 1.02 and 0.93, respectively. So, at power = 0.80 (beta = 0.20) and alpha = 0.05, the sample size in our study was equal or more than 15 for each group.

### Statistical analysis

Utilizing the statistical program for social sciences, version 23.0, the recorded data was examined (SPSS Inc., Chicago, Illinois, USA). In terms of the quantitative data, mean ± standard deviation and ranges were reported. Qualitative variables were also shown as percentages and numbers.

- *Independent samples t test* of significance was used when comparing between two means and *Mann Whitney z test*: for two-group comparisons in non-parametric data.
- *Paired sample t test* of significance was used when comparing between related sample and comparison between differences by time for non-parametric data using the *Wilcoxon Signed-Rank Sum test*.

## Results

After diet and exercise intervention, in group A, a highly significant decrease in all anthropometric parameters was observed, (*p* < 0.001), except for W/H ratio which showed no significant difference, (*p* > 0.05) (Table 2). Furthermore, a highly significant decrease in cholesterol and LDL after diet and exercise intervention among obese adolescence in group I was observed (*p* < 0.001). Whereas, a significant decrease in TG was observed (*p* < 0.05). On the other hand, a statistically significant increase in HDL was observed (*p* < 0.05) (Table 3).

**Table 2:** Comparison of anthropometric parameters before and after diet and exercise intervention in group A

Anthropometric parameters		Before	After	% of change	Test value	*p*-value
Weight (kg)	Mean ± SD	97.99 ± 15.91	88.46 ± 15.17	−9.83 ± 2.84	*z* = −3.409	<0.001
	Range	81.5–135.1	71.5–122			
Triceps SFT (mm)	Mean ± SD	30.67 ± 6.91	25.73 ± 7.58	−17.33 ± 10.42	*z* = −3.432	<0.001
	Range	17–44	11–38			
Biceps SFT (mm)	Mean ± SD	19.73 ± 7.61	15.77 ± 6.63	−20.27 ± 11.68	*z* = −3.426	<0.001
	Range	8–30	6–25			
Subscapular SFT (mm)	Mean ± SD	29.33 ± 6.85	25.80 ± 7.06	−12.65 ± 6.57	*z* = −3.284	<0.001
	Range	18–40	16–38			
Suprailiac SFT (mm)	Mean ± SD	33.00 ± 11.18	27.60 ± 9.33	−15.90 ± 6.17	*z* = −3.419	<0.001
	Range	13–50	12–40			
Abdominal SFT (mm)	Mean ± SD	42.07 ± 9.19	35.67 ± 7.69	−15.10 ± 3.79	*z* = −3.440	<0.001
	Range	30–60	25–50			
MUAC (cm)	Mean ± SD	36.00 ± 3.40	31.13 ± 3.18	−13.42 ± 5.31	*t* = −8.976	<0.001
	Range	31–43	25–39			
Waist circumf. (cm)	Mean ± SD	101.47 ± 12.88	93.33 ± 11.04	−7.88 ± 2.17	*t* = −7.845	<0.001
	Range	82–128	78–112			
Hip circumf. (cm)	Mean ± SD	119.87 ± 11.10	108.20 ± 11.78	−9.77 ± 4.17	*t* = −9.551	<0.001
	Range	107–150	88–140			
W/H ratio	Mean ± SD	0.87 ± 0.09	0.85 ± 0.09	−2.29 ± 5.57	*t* = −1.552	0.143
	Range	0.74–1.04	0.74–1.06			
BMI (wt/(ht)^2^)	Mean ± SD	36.75 ± 6.48	32.63 ± 5.83	−10.87 ± 8.81	*t* = −3.988	<0.001
	Range	29.7–52.8	25.5–45			

*SFT* skin fold thickness, *MUAC* mid-upper arm circumference, *W/H ratio* waist/hip ratio, *BMI* body mass index

**Table 3:** Comparison of laboratory parameters before and after diet and exercise intervention in group A.

Laboratory parameters		Before	After	% of change	Test value	*p*-value
Cholesterol (mg/dL)	Mean ± SD	190.07 ± 7.94	145.07 ± 17.40	-23.58 ± 9.25	*t* = −9.520	<0.001
	Range	176–200	116–177			
HDL (mg/dL)	Mean ± SD	38.40 ± 6.78	47.27 ± 5.13	23.10 ± 21.06	*t* = 2.112	0.023
	Range	30–59	35–62			
LDL (mg/dL)	Mean ± SD	131.97 ± 20.20	84.89 ± 19.94	−34.39 ± 18.51	*z* = −3.408	<0.001
	Range	107.8–174	55.2–120			
TG (mg/dL)	Mean ± SD	152.40 ± 26.42	131.93 ± 22.24	−13.43 ± 12.90	*z* = −2.553	0.029
	Range	80–187	98–188			

*HDL* high-density lipoprotein, *LDL* low-density lipoprotein, *TG* triglyceride

According to after diet, exercise and laser acupuncture intervention in group B revealed a highly significant decrease in all anthropometric parameters (except for W/H ratio which showed no significant difference, (*p* > 0.05) (*p* < 0.001) (Table 4). Moreover, it showed a highly significant decrease in cholesterol, LDL, and TG (*p* < 0.001). On the other hand, HDL showed a statistically highly significant increase, which was observed (*p* < 0.001) (Table 5).

**Table 4:** Comparison of anthropometric parameters at baseline and after diet, exercise, and laser acupuncture intervention in group B

Anthropometric parameters		Before	After	% of change	Test value	*p*-value
Weight (kg)	Mean ± SD	104.31 ± 20.05	87.98 ± 19.81	−15.66 ± 2.40	*z* = −3.221	<0.001
	Range	77–167.3	66–113			
Triceps SFT (mm)	Mean ± SD	27.77 ± 7.04	23.25 ± 6.49	−16.76 ± 4.91	*z* = −4.819	<0.001
	Range	16–38	12–34			
Biceps SFT (mm)	Mean ± SD	23.07 ± 6.58	18.97 ± 5.92	−18.35 ± 7.34	*z* = −4.837	<0.001
	Range	11–36	6–31			
Subscapular SFT (mm)	Mean ± SD	24.27 ± 7.41	19.87 ± 6.82	−18.25 ± 12.46	*z* = −4.296	<0.001
	Range	15–40	11–34			
Suprailiac SFT (mm)	Mean ± SD	34.20 ± 5.45	27.51 ± 5.56	−19.82 ± 6.80	*z* = −4.808	<0.001
	Range	25–46	20–41			
Abdominal SFT (mm)	Mean ± SD	43.37 ± 6.75	35.29 ± 6.25	−18.75 ± 6.55	*z* = −4.792	<0.001
	Range	24–55	17–47			
MUAC (cm)	Mean ± SD	36.32 ± 3.08	29.92 ± 3.66	−17.73 ± 5.51	*t* = −18.54	<0.001
	Range	32–41	22–37			
Waist circumf. (cm)	Mean ± SD	102.40 ± 16.01	92.52 ± 16.43	−10.07 ± 3.02	*t* = −19.35	<0.001
	Range	40–147	30–140			
Hip circumf. (cm)	Mean ± SD	119.93 ± 18.23	108.77 ± 18.35	−9.79 ± 4.45	*t* = −21.71	<0.001
	Range	43–148	30–138			
W/H ratio	Mean ± SD	0.86 ± 0.06	0.85 ± 0.07	−0.22 ± 3.57	*t* = −0.302	0.764
	Range	0.74–1.01	0.7–1.01			
BMI (wt/ (ht)^2^)	Mean ± SD Range	38.23 ± 6.20 30–52.6	31.38 ± 5.91 26–41	−17.92 ± 4.44	*t* = −4.291	<0.001

*SFT* skin fold thickness, *MUAC* mid-upper arm circumference, *W/H ratio* waist/hip ratio, *BMI* body mass index

**Table 5:** Comparison of laboratory parameters before and after diet, exercise, and laser acupuncture intervention in group B

Laboratory parameters		Before	After	% of change	Test value	*p*-value
Cholesterol (mg/dL)	Mean ± SD	184.20 ± 13.93	132.13 ± 25.55	−28.10 ± 13.52	*t* = 10.863	<0.001
	Range	150–200	75–183			
HDL (mg/dL)	Mean ± SD	37.80 ± 4.68	52.41 ± 7.15	38.65 ± 26.43	*t* = 5.221	<0.001
	Range	30–46	37–65			
LDL (mg/dL)	Mean ± SD	158.05 ± 36.93	70.75 ± 24.48	−53.91 ± 16.25	*z* = -4.782	<0.001
	Range	90–251.4	33.6–120			
TG (mg/dL)	Mean ± SD	157.23 ± 36.18	125.43 ± 17.73	−14.32 ± 29.57	*z* = -3.374	<0.001
	Range	82–220	90–157			

*HDL* high-density lipoprotein, *LDL* low-density lipoprotein, *TG* triglyceride

Regarding the percentage of change between group A and group B, Table 6 shows a significant difference, where the highest values were found in group B compared to group A, in the following parameters: weight, BMI, subscapular skin fold thickness (SFT), mid-upper arm circumference (MUAC), and waist circumference (*p* < 0.05). On the contrary, no significant difference was found between group A and group B, regarding the percentage of change in the following parameters: triceps SFT, biceps SFT, suprailiac SFT, abdominal SFT, hip circumference, and W/H ratio, with *p*-value > 0.05.

**Table 6:** Comparison of anthropometric parameters between group A and group B regarding the mean difference and percentages of change

Anthropometric parameters		Group I (*n* = 15)	Group II (*n* = 30)	*z*-test	p-value
Height (cm)	Mean ± SD	163.80 ± 8.18	165.30 ± 8.55	*F* = 0.370	0.693
	Range	149–176	150–184		
Weight (kg)	Mean diff.	−9.53 ± 3.62	−16.33 ± 3.24	−3.355 −2.011	0.033 0.032
	Change (%)	−9.83 ± 2.84	−15.66 ± 2.40		
Triceps SFT (mm)	Mean diff.	−4.93 ± 2.12	−4.51 ± 1.40	−0.695 −0.590	0.487 0.555
	Change (%)	−17.33 ± 10.42	−16.76 ± 4.91		
Biceps SFT (mm)	Mean diff.	−3.97 ± 2.83	−4.10 ± 1.92	−1.166 −0.193	0.243 0.847
	Change (%)	−20.27 ± 11.68	−18.35 ± 7.34		
Subscapular SFT (mm)	Mean diff.	−3.53 ± 1.00	−4.40 ± 1.79	−2.034 −3.063	0.041 0.002
	Change (%)	−12.65 ± 6.57	−18.25 ± 12.46		
Suprailiac SFT (mm)	Mean diff.	−5.40 ± 2.90	−6.69 ± 2.34	−1.755 −1.879	0.079 0.060
	Change (%)	−15.90 ± 6.17	−19.82 ± 6.80		
Abdominal SFT (mm)	Mean diff.	−6.40 ± 2.41	−8.07 ± 3.01	−1.830 −1.655	0.067 0.098
	Change (%)	−15.10 ± 3.79	−18.75 ± 6.55		
MUAC (cm)	Mean diff.	−4.87 ± 2.10	−6.39 ± 1.89	−2.511 −2.577	0.012* 0.010
	Change (%)	−13.42 ± 5.31	−17.73 ± 5.51		
Waist circumf. (cm)	Mean diff.	−8.13 ± 4.02	−9.88 ± 2.80	−2.159 −2.076	0.031 0.037
	Change (%)	−7.88 ± 2.17	−10.07 ± 3.02		
Hip circumf. (cm)	Mean diff.	−11.67 ± 4.73	−11.16 ± 2.82	−0.391 −0.217	0.696 0.828
	Change (%)	−9.77 ± 4.17	−9.79 ± 4.45		
W/H ratio	Mean diff.	0.02 ± 0.05	0.00 ± 0.03	−1.639 −1.685	0.101 0.092
	Change (%)	−2.29 ± 5.57	−0.22 ± 3.57		
BMI (wt/(ht)^2^)	Mean diff.	−4.11 ± 3.99	−6.85 ± 1.83	−2.013 −2.683	0.049 0.037
	Change (%)	−10.87 ± 8.81	−17.92 ± 4.44		

*SFT* skin fold thickness, *MUAC* mid-upper arm circumference, *W/H ratio* waist/hip ratio, *BMI* body mass index

Furthermore, there was a significant difference between group A and group B regarding the percentage of change, where the highest values were found in group B compared to group A, in the following parameters: HDL and LDL (*p* < 0.05), whereas TG and cholesterol showed no statistically significant difference (*p* > 0.05) (Table 7). So, the laser acupuncture has better effect on management of obesity in adolescents (anthropometric parameters and lipid profile).

**Table 7:** Comparison of laboratory parameters between group A and group B regarding the mean difference and percentage of change

Laboratory parameters		Group I (*n* = 15)	Group II (*n* = 30)	*z*-test	*p*-value
Cholesterol (mg/dL)	Mean diff.	−45.00 ± 18.31	−52.07 ± 26.25	−0.819	0.413
	Change (%)	−23.58 ± 9.25	−28.10 ± 13.52	−1.144	0.253
HDL (mg/dL)	Mean diff.	8.87 ± 4.12	14.62 ± 7.98	3.365	0.036
	Change (%)	23.10 ± 21.06	38.65 ± 26.43	3.288	0.038
LDL (mg/dL)	Mean diff.	−47.08 ± 28.27	−87.30 ± 37.05	−3.371	<0.001
	Change (%)	−34.39 ± 18.51	−53.91 ± 16.25	−3.227	<0.001
TG (mg/dL)	Mean diff.	−20.47 ± 12.53	−31.80 ± 22.80	−4.112	<0.001
	Change (%)	−13.43 ± 12.90	−14.32 ± 29.57	−1.769	0.096

*HDL* high-density lipoprotein, *LDL* low-density lipoprotein, *TG* triglyceride

## Discussion

Our aim was to compare the effect of diet and exercise on anthropometric parameters and lipid profile with laser acupuncture effect on obese adolescents. We found a significant decrease in all anthropometric parameters and lipid profile after the intervention in group A (LCD + exercise) and group B (LCD+ exercise+ LA) with a significant difference between both groups in weight, BMI, subscapular SFT, MUAC, and waist circumference, where the highest reduction was found in group B compared to group A but showed no statistically significant difference in W/H ratio, between the two groups. Moreover, our study revealed a significant increase in HDL serum levels in group B than group A. This indicates that the use of laser for 2 times/week for 12 weeks with diet control and exercise had a better effect on anthropometric parameters and lipid profile. We used a low-level laser as it was useful for reaching deep acupuncture points. Energy density is the most crucial variable in the laser acupuncture procedure. It has been observed that doses of 0.5–2.5 J/cm^2^ are useful for stimulating superficial acupuncture points, whereas 2.5–5.0 J/cm^2^ are beneficial for stimulating deeper sites and myofascial trigger points [14].

Our results were in agreement with a study done in 2020, which carried out a study on 38 adults divided into 2 groups: the treatment group (laser acupuncture + LCD) and the control group (sham laser acupuncture + LCD) 3 times/week for 4 weeks. The study revealed that BMI, after 12 sessions of treatment, showed a significant decrease between pre and post intervention in the treatment group and showed a significant difference between the two groups, where the highest reduction was found in group I compared to group II. This clarifies the role of laser intervention in weight loss [15]. Similarly, in 2017, a study carried out a systematic review on laser acupuncture (LA) effectiveness and proved that LA therapy had a positive effect on body weight, BMI, WC, HC, and fat percentage [16]. Furthermore, in (2015), another study found a positive effect of laser acupuncture therapy combined with low calorie diet + exercise training in obese patients. Those results showed a statistically significant decrease in BMI, waist circumference, abdominal circumference, and total cholesterol levels in this group compared to the low calorie diet + exercise group [17]. In disagreement with our results, in (2020), a study found a statistically significant decrease in waist/hip ratio in the treatment group (LA + LCD) compared to the control group (sham laser acupuncture + LCD) [15]. In agreement with our result, in 2014, a research was done on 76 adult females classified into 3 groups: low-calorie diet with exercise (moderate) 30 min/day, 3d/week for 12 weeks; needle acupuncture with LCD + exercise; and laser acupuncture with LCD + exercise. Nutritional intervention showed a highly significant improvement in the anthropometric measurements, in the form of decrease in body weight, BMI, WC, and hip C. Whereas, laser acupuncture with low calorie diet (LCD) + exercise intervention had higher significant decrease values of body anthropometry in (body weight, BMI, WC, hip C, and WHR) and some parameters of lipid profile (decreased total cholesterol and LDL). And by comparison between the two groups, they found that the combination between LCD + exercise + LA has better effect on anthropometric parameter and lipid profile [18]. On the contrary to our results, the study conducted in 2020 revealed no significant differences in HDL levels between the laser-treatment group and the control group (LCD + sham LA) [15]. However, this might be attributed to their short period of treatment of only 4 weeks, with laser power 50 mw and density 4 J only. Whereas, our study patients have undergone 2 sessions/week for 12 weeks with laser power = 100 mw and density = 6 J/cm^2^. In accordance with our results, in 2020, a study detected that triglyceride levels showed a statistically significant decrease post intervention in the laser + low-calorie diet group and the control group (LCD + sham LA), although he found no significant difference between the two groups [18].

Similarly, in 2015, a study concluded that needle acupuncture for 3–6 months (2 sessions weekly) in combination with a low-caloric diet showed a highly significant decrease in weight, BMI, fat percentage, fat mass, and lipid profile (cholesterol and triglyceride). These findings are compatible with those concluded by our study. On the contrary to our findings, the same study showed no significant difference in HDL and LDL [19]. However, this disagreement can be attributed to the fact that, in our methodology, we used exercise in combination with a low-calorie diet and laser acupuncture. Moreover, various factors were reported to affect HDL level, e.g., food and exercise. In addition, exercise could increase HDL levels up to 25% [18].

Traditional medical theory denotes that acupuncture stimulates peripheral nerves at acupoints, which then affects the central nervous system. In contrast to exercise and food alone, which had little influence on this alteration, signals are then transmitted by activated nerves, changing satiety and mood [15, 20].

It has been shown that the application of electroacupuncture at ST-36 and ST-44 could inhibit the hyperactivity of the stomach induced by the increase in the electrical activity stimulation of the lateral hypothalamic area (LHA), leading to the activation of the satiety center indicating that acupuncture has an anticholinergic function, through which β receptors were activated to inhibit appetite and eliminate hunger [15, 21].

Stimulation of the QuChi (LI 11) and Tianshu (St 25) body acupuncture points had a regulatory effect on intestinal motility, whereas the stimulation of Zusanli (St 36) and Neiting (St 44) increases excitability of the satiety center in the ventral medial nucleus of the hypothalamus [21]. So we use these points in our study.

Acupuncture also affects the immune system and metabolism in a lipolytic way, according to a review by Cabyoglu et al. The findings indicated that body acupuncture was more successful in reducing waist and hip circumferences than other methods, which indicates that more abdominal adipose tissue was lost, resulting in the creation of fewer inflammatory markers for immune system stimulation [22].

In our study, we used laser over the acupoints which enhanced the effect by biostimulation and was more safe and painless.

Laser acupuncture controls obesity producing a therapeutic effect by reducing both BW and BMI and by inducing physiological changes improving quality of life by reducing appetite, improving mood resulting in weight loss maintenance. Furthermore, subjects demonstrate good compliance [23]. So laser treatment has an additive effect with diet and exercise.

## Conclusion

Laser acupuncture is effective in the management of obesity, as it added an extra effect during the use of diet and exercise. It was safe, easy to use, and painless which was good for treating obese children with no fear and no pain. LA gave good effect in decreasing the anthropometric parameters and lipid profile.
