# Correction to: Effects of laser acupuncture on anthropometric parameters and lipid profile in obese adolescents

**DOI:** 10.1007/s10103-023-03889-w

**Published:** 2023-09-25

**Authors:** Rasha Zohdy, Jehan Alsharnoubi, Wafaa Kandeel, Maha Saber, Hanaa Reyad Abdallah Elmorsy, Ola Dabbous

**Affiliations:** 1 Biological Anthropology, Medical Research and Clinical Studies Institute, National Research Center, Cairo, Egypt; 2 Pediatrics, National Institute of Laser Enhanced Sciences (N.I.L.E.S), Cairo University, Cairo, Egypt; 3 Biological Anthropology, Medical Research and Clinical Studies Institute, National Research center, Cairo, Egypt; 4 Childhood Health Complementary Medicine Department, Medical Research and Clinical Studies Institute, National Research Center, Cairo, Egypt; 5 Medical Application of Laser, National Institute of Laser Enhanced Sciences (N.I.L.E.S), Cairo University, Cairo, Egypt

**Correction to: Lasers Med Sci**

10.1007/s10103-023-03861-8

In the published paper, the author Hanaa Reyad Abdallah Elmorsy was missed to be included as part of the author group.

The original article has been corrected.

